# Film dosimetry for occupant exposure monitoring within Far‐UVC installations

**DOI:** 10.1111/php.13960

**Published:** 2024-05-03

**Authors:** David Welch, Raabia Hashmi, Camryn Petersen, Steven Erde, David J. Brenner, Edward Nardell

**Affiliations:** ^1^ Center for Radiological Research Columbia University Irving Medical Center New York New York USA; ^2^ College of Dental Medicine Columbia University Irving Medical Center New York New York USA; ^3^ Division of Global Health Equity, Brigham & Women's Hospital Harvard Medical School Boston Massachusetts USA

**Keywords:** exposure monitoring, far‐UVC, film dosimetry

## Abstract

Far‐UVC radiation between 200 and 230 nm is a promising technology for reducing airborne disease transmission. Previous work with far‐UVC lamps has demonstrated the efficacy of far‐UVC radiation to inactivate bacteria and viruses while presenting minimal human health hazards. While far‐UVC intentionally exposes the occupied space, effectively disinfecting air between occupants, installations must still ensure that occupant eye and skin exposure is within the recommended daily limits. This study examines far‐UVC‐sensitive films for measuring the dose received by occupants within two real‐world far‐UVC installations. The film is characterized for accuracy, angular response, wavelength response, and sources of uncertainty in film response, and used to obtain individual exposure doses that account for both the non‐uniform irradiance and the unique motion of individuals within the space. Dosimetry results using the films, which account for the time‐weighted average exposure of an occupant, ranged from 10% to 49% of the maximum calculated stationary dose based on peak irradiance measurements. Results from this study spotlight the need to incorporate time‐weighted average considerations into the design and safety assessment of far‐UVC installations to ultimately operate far‐UVC technology with its full potential to prevent the spread of potentially fatal infectious diseases.

## Graphical abstract

Far‐UVC radiation between 200 and 230 nm is a promising technology for reducing airborne disease transmission. This study examines far‐UVC‐sensitive films for measuring the dose received by occupants within two real‐world far‐UVC installations.

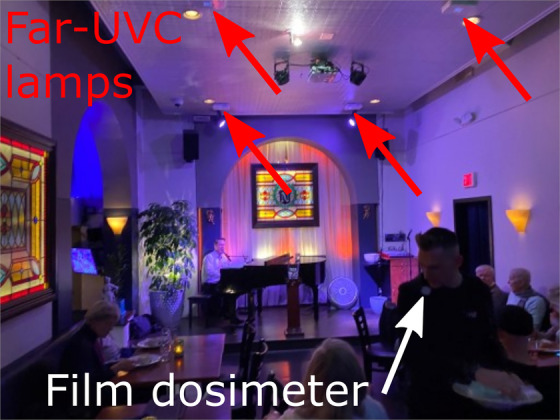

**Abbreviations**
ACGIH: American Conference of Governmental Industrial Hygienists
ACH: air changes per hour
ASHRAE: American Society of Heating, Refrigerating and Air‐Conditioning Engineers
CD: color density
CI: confidence interval
CoV‐2: Severe acute respiratory syndrome coronavirus 2
eACH: equivalent air changes per hour
netCD: net color density
PI: prediction interval
SARS‐CoV‐2: Severe acute respiratory syndrome coronavirus 2
TB: tuberculosis
TLV: Threshold Limit Value
UL: Underwriters Laboratory
UV: ultraviolet
UVC: ultraviolet radiation c

## INTRODUCTION

Nearly 20 years ago, in response to a resurgence of tuberculosis (TB) in the United States and other resource‐rich countries, there was renewed interest in germicidal UV air disinfection—an old technology thought to be unnecessary after the advent of vaccines and antibiotics. A similar surge in interest in germicidal UV has occurred in response to the SARS‐CoV‐2 (CoV‐2) pandemic.^1^ Both TB and CoV‐2 are airborne respiratory pathogens for which air disinfection can reduce transmission.^2^ In addition to the application of the long‐available upper room 254 nm UV, generated by mercury lamps, the more recently developed 265–270 nm upper‐room LED source UV^3^ and whole room 222 nm UV,^4^, ^5^, ^6^, ^7^ generated by filtered krypton chloride lamps,^8^ promise to be even more effective and safer for room occupants. Recently approved American Society of Heating, Refrigerating and Air‐Conditioning Engineers (ASHRAE) ventilation standards for airborne infection control^9^ propose ventilation levels, or equivalent ventilation levels, that can only be achieved practically and sustainably through germicidal UV air disinfection. High levels of equivalent ventilation are especially important for mitigating spread from super spreaders, believed to account for a substantial portion of airborne respiratory infections.^10^

UV exposure of room occupants is limited by its potential for skin and eye irritation and damage.^11^ For both upper‐room and whole‐room UV safety, official recommendations have interpreted the well‐established ACGIH (originally American Conference of Governmental Industrial Hygienists) 8‐h threshold limit value (TLV) eye exposure limits,^12^ adjusted for each germicidal wavelength, as if room occupants stared continuously for 8 h at the lamp at the point of peak irradiance in the room, measured at 6.5 to 7 ft (1.98 to 2.1 m) off the floor, the eye height of an unusually tall standing room occupant. The problem is that this “worst case” approach to occupant safety from a UV perspective greatly limits the germicidal UV dose throughout the room volume thereby reducing efficacy and increasing the infection risk of room occupants. This ultraconservative approach to UV fixture safety, ignoring real‐world time–motion exposure data,^13^ has been continued by the recent Underwriters Laboratory certification testing process for fixtures. Whereas workers and others have become infected and have died from exposure to TB, CoV‐2, and other airborne respiratory infections, to our knowledge there have been no reports of serious eye or skin damage due to UV overexposure in hundreds of thousands of professionally installed fixtures, which is rare in any case,^14^ and entirely preventable without constraining germicidal UV efficacy.

In a 2005 study of human exposure to 254 nm upper‐room germicidal UV, Nardell and colleagues hypothesized that actual measured room occupant exposure (time‐weighted average) would be only a small fraction of what peak level irradiance at 6.5 to 7 ft (1.98 to 2.1 m) would predict.^15^ Using a portable tracking UV meter worn around the neck of 19 occupants, the highest measured dose (2 mJ/cm^2^) was only one‐third of the 8 h TLV, and the next highest doses extrapolated to 8 h were 21%, 18%, 17%, 15%, and 14% of the TLV. The highest ratio of measured dose to the dose calculated from the peak irradiance was 31%. Subjects included patients, nurses, teachers, office workers, and shelter workers. In the present study, we take a similar approach to monitoring actual time and motion exposure of room occupants in two different 222 nm UV settings through shoulder‐level film‐badge UV‐exposure monitoring. We propose that shoulder‐level film monitoring may provide a practical method of on‐site estimation of both breathing zone efficacy (calculated equivalent air changes per hour) and safety for exposed head and neck skin. While it is difficult to simulate eye exposure with portable meters or film badges, for most overhead 222 nm UV applications, the eyes are well protected by the brow and eyelids.^16^, ^17^ Early UV eye irritation is readily sensed without any significant eye damage since corneal epithelial cells are normally sloughed and replaced every 48 h.^18^ The absence of eye symptoms in combination with shoulder‐level monitoring doses within the TLV provides practical assurance of UV safety while allowing for optimal air disinfection efficacy.

## MATERIALS AND METHODS

### UV radiation characterization

Spectral characterization of the far‐UVC fixtures was performed using a Gigahertz Optik BTS2048‐UV spectroradiometer (Gigahertz‐Optik Inc, Amesbury, MA). A UIT2400 meter (Ushio America Inc., Cypress, CA, USA) equipped with a SED220 detector was used for irradiance measurements.

### UV radiation‐sensitive films

The film product used in this study is OrthoChromic Film OC‐1 (Orthochrome Inc., Hillsborough, NJ). OC‐1 film is marketed as a tool for radiation therapy measurements of ionizing radiation though its utility in UVC dosimetry has been demonstrated previously.^16^, ^19^ The flexible film is 155 μm thick, consisting of a 30 μm active coating on a 125 μm white polyester base. The active region must be oriented toward the UV source during measurements since the polyester layer is opaque. Importantly, the film is minimally sensitive to room lighting conditions and the limited exposure times of this experiment have minimal impact on the dose measured.^19^ The active layer of the film changes color upon exposure to radiation and no processing is required before color quantification.

An Epson Perfection V850 Pro photo flatbed scanner (Epson, Suwa, NGN, Japan) was utilized for quantification of the color of each film. The scanner was operated in reflection mode and captured 48‐bit RGB TIFF images with all color correction factors turned off. MATLAB (Mathworks, Natick, MA) was used for analysis. The red color channel was utilized for this study. The color density (CD) of each pixel value was calculated with the equation:

(1) $$\mathrm{CD}=-\log_{10}\left(\frac{\text{pixel value}}{65536}\right),$$

and the net color density (netCD) was calculated as:

(2) $$\text{netCD}=\mathrm{CD}-{\mathrm{CD}}_{\text{unexposed}}=d_{x}\left(D\right),$$

with the CD_unexposed_ being the *CD* for an unexposed piece of film and *d*_*x*_(*D*) being the response for a given radiant exposure dose (*D*). Data for each exposure condition were matched to a fitting function with the form:

(3) $$d_{x}\left(D\right)=\frac{a+\mathrm{bD}}{D+c},$$

where *a*, *b*, and *c* are constants. The fitting function was optimized in MATLAB using the curve fitting tool to minimize the squared difference between the experimental data and the fit equation.

### Film characterization: dose–response accuracy

A calibration curve for each of the far‐UVC lamps was generated by exposing pieces of film to a range of radiant exposure doses. Exposures were performed with the lamp oriented normally to the film. The UIT2400 irradiance meter was positioned adjacent to exposed film and recorded the total radiant exposure upon a given piece of film. The irradiance at the meter position during exposures was confirmed to agree with the irradiance at the film position. Films were processed to determine the netCD and plotted for their respective dose. After fitting to Equation 3, the 95% confidence interval (CI) was found using the MATLAB *predint* function for a nonsimultaneous bound type on the fitted curve. The CI bounds for the fit are defined as

(4) $$\mathrm{CI}=y\pm t\sqrt{S}$$

where *y* is the netCD value produced by the fit curve for a given dose, *t* is computed from the Student's *t* cumulative distribution function for a 95% (α = 0.05), and *S* is the estimated variance. Similarly, the 95% prediction interval (PI) used the *predint* function for a nonsimultaneous bound for a new observation was calculated using;

(5) $$\mathrm{PI}=y\pm t\sqrt{\mathrm{MSE}+S}$$

where MSE is the mean‐squared error. The normalized relative widths were computed by dividing the width of the 95% CI and PI interval at each dose by the dose value.

### Film characterization: angular response

Film was exposed at angles from 0° (normal to the lamp) to 90° using an optically filtered KrCl lamp (Vive, R‐Zero Systems, Inc., South Salt Lake, UT). All films were exposed to a dose of 10 mJ/cm^2^ as measured by the UIT2400 meter. The mean dose measured by the film at each exposure dose was recorded, and the resulting doses were normalized by dividing by the dose at 0° incidence. The angular response was compared to a cosine response by dividing the normalized dose at each angle by the cosine of that angle.

### Film characterization: spectral response

The spectral sensitivity of the film was examined using an optical system to enable monochromatic UVC exposures to the film. An EQ‐77 Laser‐Driven Light Source (Energetiq Technology, Inc., Wilmington, MA) provided a high‐brightness broadband output across the wavelength range of 170–2500 nm. A pair of off‐axis parabolic mirrors focused the EQ‐77 output into a Cornerstone 260 1/4 m monochromator (CS260‐RG‐2‐FH‐A, Newport Corporation, Irvine, CA). The monochromator was equipped with a 1201.6 g/mm plane blazed holographic reflection grating (#53‐*‐200H with master no. 5482, 250 nm nominal blaze wavelength, Newport) to maximize optical throughput in the UVC. Fixed slits with a slit size of 600 μm (77216, Newport) were used for all experiments. With a 600 μm slit width and the 1201.6 g/mm grating, the theoretical resolution of the monochromator was 1.9 nm. The measured full width at half maximum was between 2.0 and 2.2 nm for all peak wavelengths used in this study.

The output of the monochromator was used to expose the film to a range of doses for center wavelengths ranging from 200 to 300 nm in 10 nm increments. Exposures using wavelengths from 230 to 270 nm utilized an OD 1.0 neutral density filter (FRQ‐ND10, Newport Corporation) in the path of the output to reduce the output power and therefore permit a suitable range of doses to be evaluated to these wavelengths. The irradiance upon the film at each wavelength was determined by recording the output power on a UV‐enhanced silicone photodetector (818‐UV/DB, Newport) with an 843‐R optical power meter (Newport) and dividing by the exposed area of the film found by scanning the film and computing area using ImageJ (National Institute of Health). The best‐fit equation (Equation 3) was determined for each of the exposure wavelengths examined.

### Film characterization: sources of uncertainty

Uncertainty in the film response was explored by measuring contributions of room light exposure to film color change, changes to the film color dependent on time of analysis post‐exposure, and additional exposure and color change due to the illumination during the film scanning process.

Room lighting effects were tested by exposing films for up to 8 h within our laboratory space with only the normal visible room lighting upon the films. Films were exposed in three different locations in the lab at a height of about 1.4 m to approximate shoulder height. The room has a ceiling height of 2.7 m and fluorescent lights are within recessed ceiling troffers. Films were scanned using the normal film processing protocol and analyzed for changes in netCD.

The possibility of films continuing to change color in the time postexposure was examined by re‐scanning films at times following their initial analysis. Eighty‐five films were randomly selected for analysis from previous experiments using the film in our lab. The time between the initial analysis scan and the second scan ranged from 15 to 407 days. The films were protected from light exposure during the time between scans. The change in netCD between the initial scan and the later scan was analyzed.

Finally, the effect of possible induced color change due to repeated optical scans was examined. An initially unexposed piece of film was scanned 10 times in succession and the color density (CD) was evaluated for each scan.

### Far‐UVC installations

**FIGURE 1:**
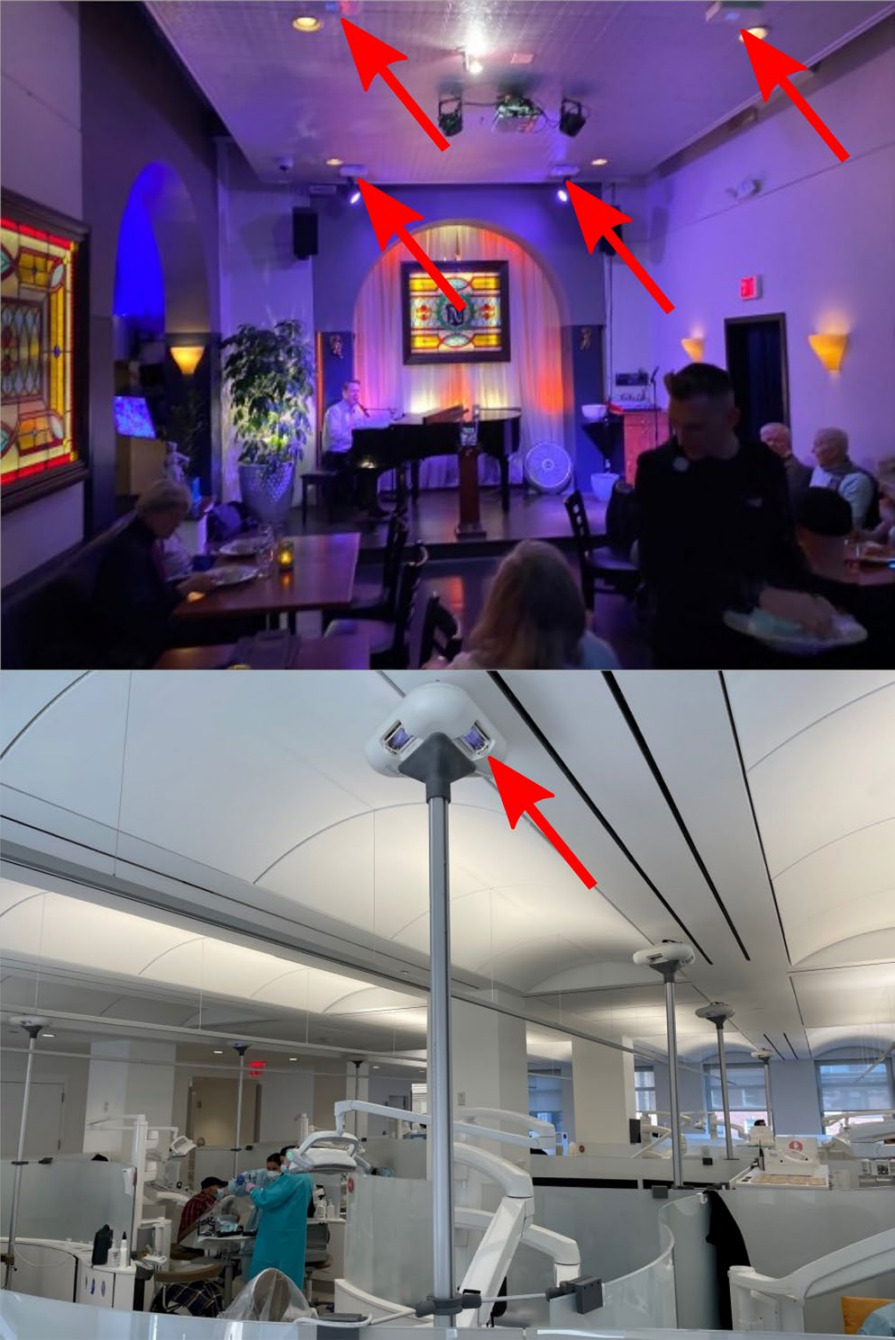
Photographs showing the Boston installation (top) and the dental clinic installation (bottom). Red arrows indicate the positions of lamps within each room.

Two locations were used for dose monitoring using films attached to occupants. Photos showing each installation are in Figure 1. The first installation was within a restaurant and club venue in Boston, MA, USA. Far‐UVC fixtures (Far UV Technologies, Inc., Kansas City, MO) containing filtered KrCl lamps emitting principally at 222 nm were installed in the ceiling emitting downward. The UV emission spectrum for these lamps is provided in the Figure S1. All the KrCl fixtures in the Boston venue are continuously emitting during operating hours. Seven lamps were located on the ceiling within the space examined in this work. Six were in the main stage and dining area (approximately 9.3 m × 4.78 m, ceiling height 3.63 m), and the remaining lamp was in a small alcove seating area off the main room. The single lamp in the alcove area was modified to include a diffuser since the ceiling height in that area was only 2 m tall.

The second installation was within the Columbia University College of Dental Medicine Teaching Clinic in New York City, NY, USA. Far‐UVC fixtures (Vive, R‐Zero Systems, Inc.) containing filtered KrCl lamps emitting principally at 222 nm were installed on custom stands throughout the room. The UV emission spectrum for these lamps is provided in Figure S1. The dental clinic is approximately 400 m^2^ with a 3 m high ceiling; 16 lamps were installed throughout the clinic on custom support poles to position the lamp at a height of 2.9 m. Each Vive unit contains 3 KrCl bulbs with optical filters, and each bulb was oriented at a 45° outward angle. All fixtures in the dental clinic were operated continuously during the periods of personal monitoring.

### Individual far‐UVC exposure monitoring

Films were affixed to occupants within each of the spaces with far‐UVC fixtures installed to monitor the far‐UVC dose received by an individual while naturally moving within the space. Films were attached to the occupants using double‐sided adhesive tape to directly attach the film to the clothing of the occupant. Occupants were instructed to attach the film to the shoulder or collar of their clothing, with the UV‐sensitive side of the film facing upward. The occupants wearing the films were then instructed to behave as they normally would within the space. After a period of exposure, typically between 1 and 5 h long, the films were returned and the total time the film was worn within the space by each occupant was recorded. Films from the Boston venue were mailed to Columbia University in New York for scanning and processing within 2 weeks following exposure. Film exposures performed at Columbia University were scanned and processed between 1 and 5 days following the exposure day.

Each film was analyzed to determine the radiant exposure, in units of mJ/cm^2^, based on the color change. The average irradiance (*I*_ave_) received upon each film was determined by dividing the dose by the recorded exposure time for each film. The average irradiance was used to determine the equivalent air changes per hour (eACH) based on the susceptibility of airborne human coronavirus OC43 using

(6) $$\text{eACH}=k\times I_{\mathrm{ave}}\times 3600$$

with *k* = 12.4 cm^2^/mJ, the experimentally determined susceptibility of human coronavirus OC43 to 222 nm exposure from Welch et al.^6^

## RESULTS

### Film characterization: dose–response accuracy

**FIGURE 2:**
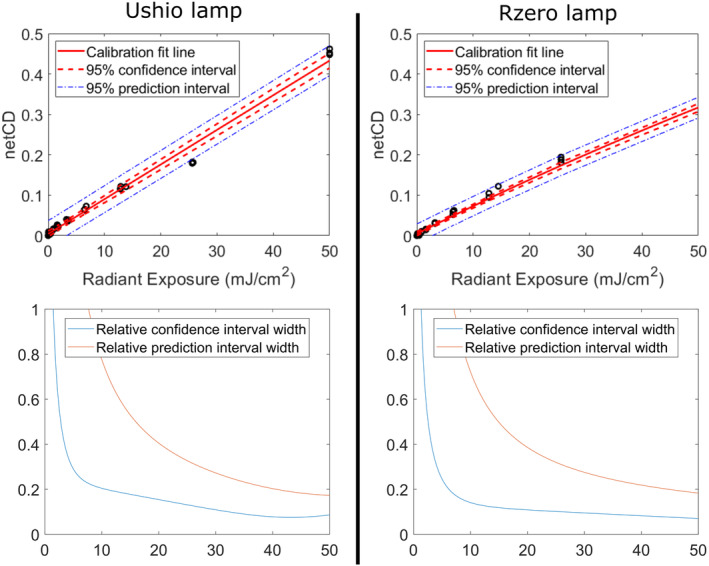
Calibration data and fit lines are plotted for the two far‐UVC lamps examined in this study. The confidence and prediction intervals are included alongside each best fit line. The lower plots show the relative widths of the confidence and prediction intervals across the dose–response calibration range.

A calibration curve specific for the film dose–response from exposure to each of the two far‐UVC fixtures was established. For the Far‐UVC fixtures used in the Boston venue, the coefficients calculated to fit Equation 3 were *a* = 0.1902 (95% CI (0.09799, 0.2825)), *b* = 0.4735 (0.4117, 0.5353), and *c* = 41.55 (33.31, 49.78), and the goodness‐of‐fit calculations produced an *r*‐squared value of 0.9973. For the R‐zero fixtures used in the Columbia Dental Clinic, the coefficients calculated to fit Equation 3 were *a* = 0.03846 (95% CI (−0.1456, 0.2225)), *b* = 0.8497 (0.5669, 1.133), and *c* = 90.87 (53.12, 128.6), and the goodness‐of‐fit calculations produced an *r*‐squared value of 0.9961. The calibration fit line for each of the lamps is provided in Figure 2. The relative CI and PI width with respect to each dose in the calibration fit for each lamp is also included in Figure 2.

### Film characterization: angular response

**FIGURE 3:**
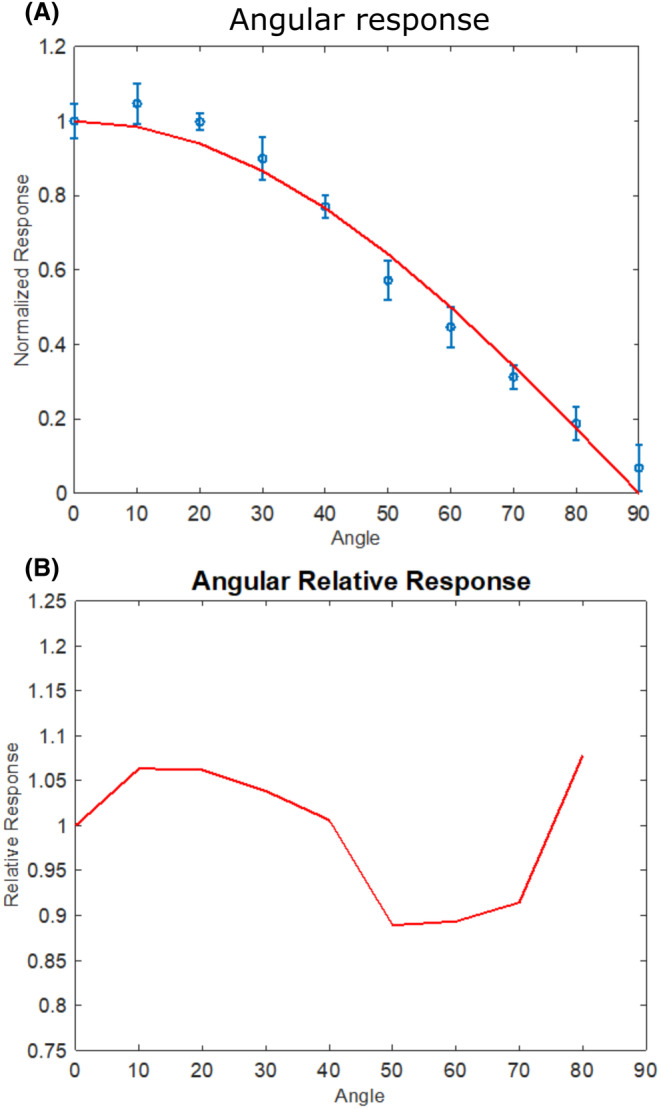
(A) The angular response of the OC‐1 film is compared to an ideal cosine response (solid line). (B) The relative response of the film compared to an ideal cosine response.

The angular response of the film was evaluated using the R‐Zero lamp, and the corresponding calibration fit was determined in this work. The normalized dose for each evaluated angle is plotted in Figure 3. Dividing the normalized dose–response by the cosine of each evaluated angle produced the relative angular response plotted in Figure 3. A perfect cosine response would yield a value of 1 across all evaluated angles.

### Film characterization: wavelength response

**FIGURE 4:**
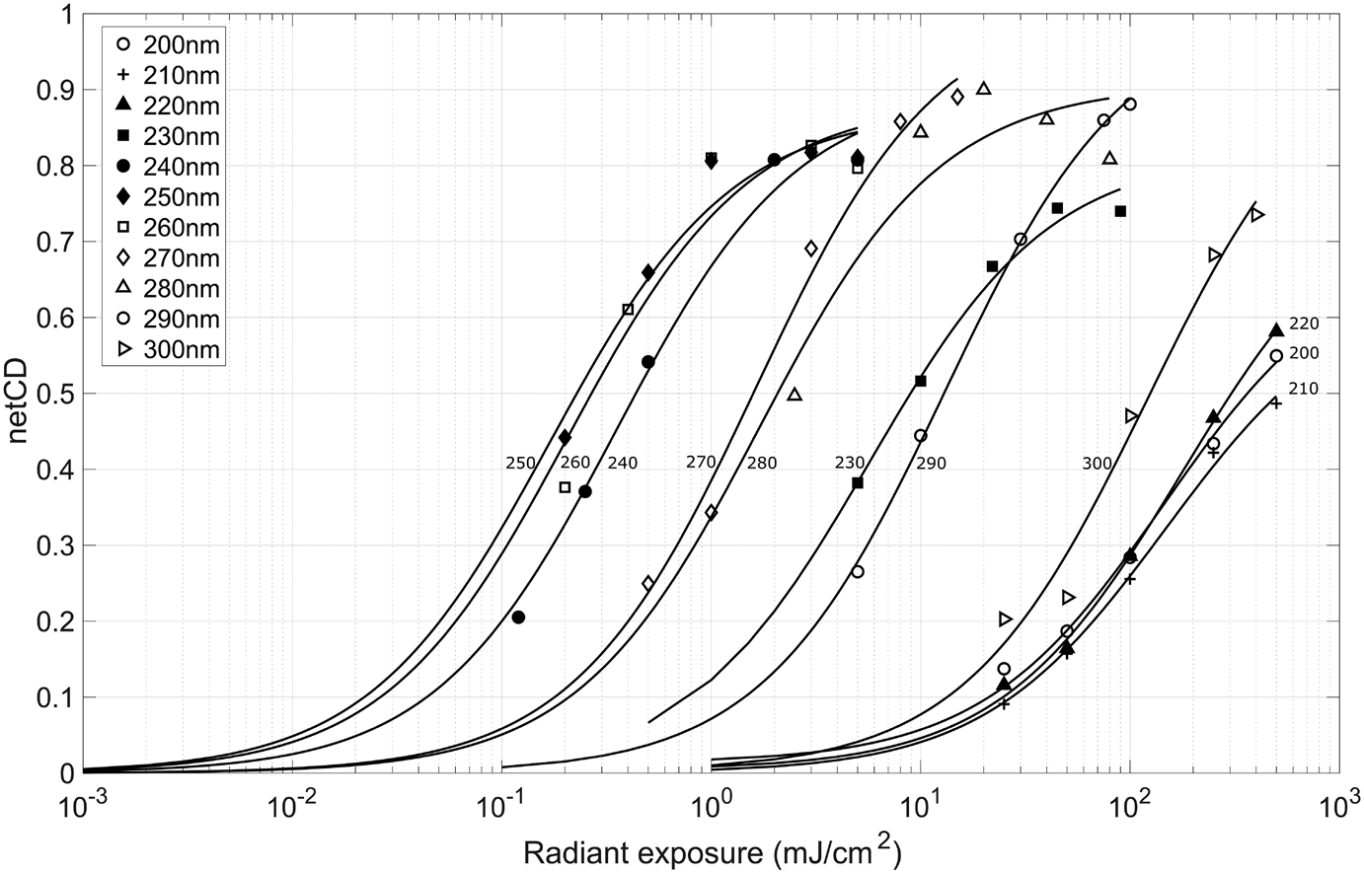
The film color change (netCD) for a range of radiant exposures is plotted for monochromatic exposures ranging from 200 to 300 nm. A best‐fit line for each wavelength is included on the plot.

**FIGURE 5:**
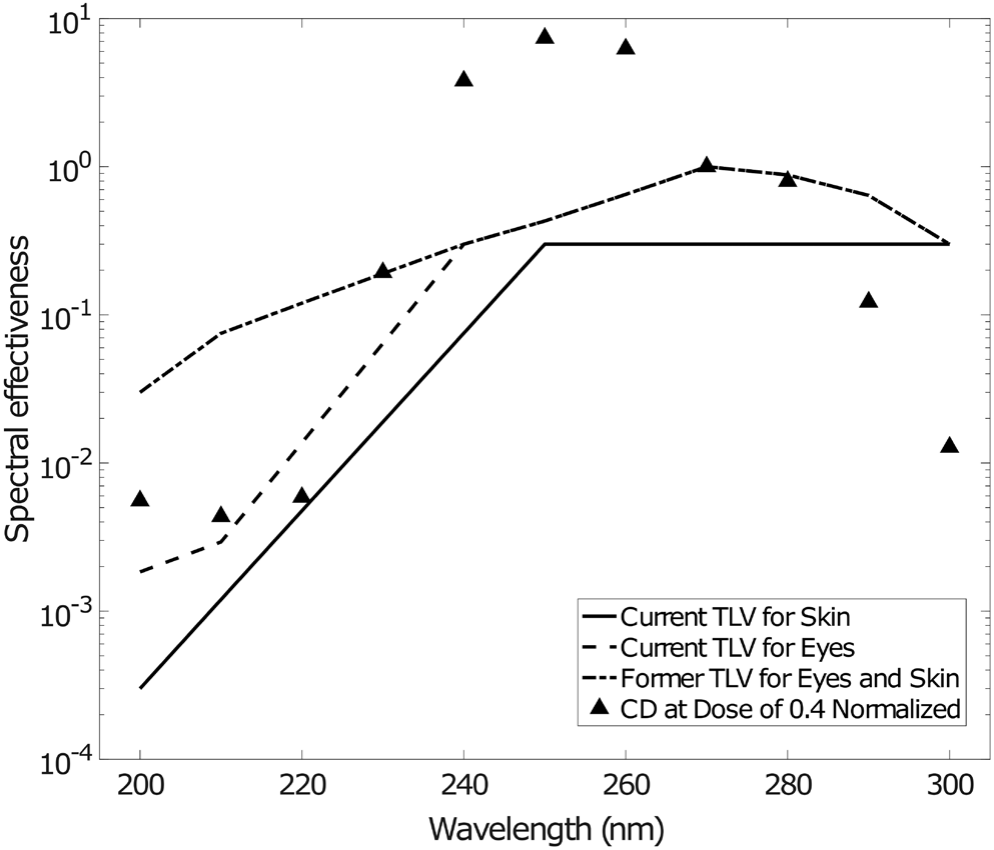
The relative spectral effectiveness of wavelengths from 200 to 300 nm. The spectral effectiveness was normalized to the response at 270 nm. Lines indicating recommended exposure limits published by the ACGIH are plotted and include lines for both values currently in effect (2022 to current) or those that were previously published (before 2022). Unlike the previous TLVs, current ACGIH TLVs have differing values for eye and skin exposure recommendations.

Calibration fit curves for each of the 11 monochromatic exposure wavelengths are plotted in Figure 4. The film was found to have a peak sensitivity at 250 nm. As with previous studies on the use of UV film dosimeters,^20^ it is useful to compare the wavelength response of this film with published spectral hazard curves. This was evaluated by determining the dose for each wavelength to produce a netCD value of 0.4 and then normalizing these values by dividing by the dose to produce a netCD of 0.4 at 270 nm. The spectral effectiveness with wavelength is plotted in Figure 5 along with the former (before year 2021) and current (year 2022 to present) spectral hazard curves recommended by the ACGIH. The 2022 ACGIH recommendations specified different hazards for skin and eye exposure, and this is reflected in the separate hazard curves.^12^

### Film characterization: sources of uncertainty

The films exposed to room lighting increased in netCD at all three positions within the room. Using a linear fit of the change in netCD value with room exposure time produces an estimated average netCD change of 0.0018 per hour across the three positions. A plot of the netCD over the 8 h for the three locations is included in Figure S2.

Results for the change in netCD for films stored up to 407 days after the initial scan are plotted in Figure S3. A linear fit of these points estimates an increase of 8 × 10^−6^ netCD per day between scans.

Results from repeated scans of a film, with only the scanner illumination exposing the film, are plotted in Figure S4. A linear fit of these results yields an estimated increase in netCD of 1 × 10^−4^ per scan.

### Individual far‐UVC exposure monitoring

Film dosimetry data were collected on multiple occasions at both the Boston venue and the Columbia Dental Clinic. The films were attached to a variety of occupants of each space. For instance, measurements within the Boston venue were made on performers who were primarily on a raised stage at the front of the room, staff who were moving about the room, as well as patrons who were likely seated for most of their visit. The results for each of the dosimetry films are included in Table 1. The table includes the exposure time for each film and the radiant exposure dose received by each film (calculated using the appropriate calibration for the lamps installed in each location). The data were also used to calculate the average irradiance upon each film and the extrapolated dose if the exposure was to be 8 h long. The final calculation included in the table is the eACH calculated using the susceptibility of human coronavirus OC43 as determined in a benchtop chamber study.^6^

**TABLE 1:** Results from personalized far‐UVC exposure monitoring with the two spaces examined in this study.

Film number	Exposure time (min)	Dose measured (mJ/cm^2^)	Average irradiance (μW/cm^2^)	Extrapolated 8‐h dose (mJ/cm^2^)	eACH (with *k* = 12.4 cm^2^/mJ)
Dental_01	60	2.07	0.57	16.53	25.6
Dental_02	68	4.96	1.22	35.04	54.3
Dental_03	155	13.22	1.42	40.93	63.4
Dental_04	135	4.64	0.57	16.50	25.6
Dental_05	114	5.87	0.86	24.70	38.3
Dental_06	119	6.47	0.91	26.08	40.4
Dental_07	115	2.55	0.37	10.66	16.5
Dental_08	141	6.95	0.82	23.65	36.7
Dental_09	144	10.45	1.21	34.85	54.0
Dental_10	118	9.18	1.30	37.35	57.9
Dental_11	178	15.03	1.41	40.53	62.8
Dental_12	66	1.90	0.48	13.84	21.5
Dental_13	188	3.76	0.33	9.59	14.9
Dental_14	151	5.71	0.63	18.14	28.1
Dental_15	197	11.68	0.99	28.45	44.1
Dental_16	178	12.84	1.20	34.62	53.7
Dental_17	147	5.96	0.68	19.45	30.1
Boston_01	287	10.46	2.68	77.21	119.7
Boston_02	289	8.65	1.19	34.30	53.2
Boston_03	65	19.07	2.65	76.28	118.2
Boston_04	121	26.06	3.62	104.24	161.6
Boston_05	120	14.21	1.96	56.37	87.4
Boston_06	120	11.61	1.61	46.45	72.0
Boston_07	121	19.55	2.71	78.19	121.2
Boston_08	120	13.93	1.93	55.71	86.4
Boston_09	120	9.26	1.29	37.03	57.4
Boston_10	120	8.25	1.20	34.45	53.4
Boston_11	120	12.30	1.71	49.20	76.3

*Note*: Exposure times and dose were determined for each film and used to calculate the average irradiance, extrapolated 8‐h dose, and eACH.

Horizontal irradiance measurements using the UIT2400 meter were also performed throughout the Boston venue at a height of 1.8 m. An irradiance of 4.2 μW/cm^2^ was measured at the position of the performer on stage, and this was the peak value throughout the main area of the installation. Extrapolating this irradiance to an 8‐h exposure would give a dose of 120 mJ/cm^2^. Also included in the far‐UVC installation area was a section of the room with lower ceilings, and a peak irradiance of 20.2 μW/cm^2^ (8‐h extrapolated dose of 581 mJ/cm^2^) was recorded at a height of 1.8 m directly under that lamp. Results for the extrapolated 8‐h dose in the Boston venue ranged from 34.30 to 104.24 mJ/cm^2^, with an average of 59.04 mJ/cm^2^. The average 8‐h dose using the films to account for the time‐weighted average exposure of an occupant was approximately 49% of the maximum stationary dose in the main room area found using the optical power meter, and only 10% of the maximum stationary dose in the section with a lower ceiling height.

The films from the Columbia Dental Clinic occupants were primarily worn by dental providers who were in the space for an extended period working with multiple patients. The providers were both sitting and standing within the space while wearing the film dosimeters. Using the optical power meter, the maximum value for horizontal irradiance at a height of 1.8 m from the floor was 3.98 μW/cm^2^, which gives an 8‐h extrapolated dose of 114 mJ/cm^2^. Results for the extrapolated 8‐h dose from individual film dosimetry monitoring in the dental clinic ranged from 9.59 to 40.93 mJ/cm^2^, with an average of 25.35 mJ/cm^2^. The average 8‐h time‐weighted exposure dose, determined using individual film dosimetry, was approximately 22% of the dose for a stationary exposure in the location of maximum irradiance found using the optical power meter.

## DISCUSSION

Installations of far‐UVC fixtures within occupied locations are becoming more common and ensuring that these installations operate within recommended exposure limits is essential for safety. However, the application of overly cautious methods of determining the potential exposure doses to occupants of the space could needlessly restrict the benefits of installing this antimicrobial technology. This study has demonstrated the use of individual monitoring of far‐UVC exposure using films attached to the shoulder for monitoring skin exposure, and this also serves as a proxy for the dose in the breathing zone of an individual for predicting efficacy. While previous studies of individual dosimetry analyzed upper‐room UVC installations which hoped to limit the dose received,^15^ this is the first occupant exposure analysis of far‐UVC installations which have been designed to intentionally expose the occupied space.

Beyond demonstrating the use of film for dose monitoring, perhaps the most important result of this study is that the dose received by individuals is only a fraction of the worst‐case scenario exposure, with values in this study of individual doses averaging from 10% to 49% of the maximum dose in the space (which assumes 8 h of continuous exposure at the maximum irradiance in the room). This result is not surprising, since one would not expect an occupant to be stationary at the highest irradiance point within a non‐uniformly exposed space. However, the range of the exposure doses which is received by occupants can help provide guidance on how TLVs should be applied when designing and installing far‐UVC fixtures. These results highlight the need for the practical usage of the space to be incorporated into the evaluation of an installation for safety. The typical position of occupants and their proportion of time spent in different areas is important to consider when the irradiance across a room varies. Evaluating a room for time‐motion does inevitably increase the burden of verifying if an installation can operate within recommended safety limits; however, a better understanding of the safety hazards will aid in designing installations that are both safe and effective at reducing disease transmission.

The measurements of average irradiance received by occupants were utilized to compute potential inactivation of a virus in the breathing zone. The average eACH in the breathing zone of monitored occupants attributed to the far‐UVC exposure was 39 for the dental clinic and 91 for the Boston installation. This level of equivalent air changes is well beyond the 12 air changes per hour (ACH) mechanical ventilation rate recommended in hospital insulation rooms.^21^ Even achieving 39 ACH using mechanical ventilation is difficult, with such a system inevitably being loud, creating an uncomfortable draft, and expensive to install and operate. While previous studies for individual UV exposure dosimetry were primarily concerned with safety, this film dosimetry approach has the dual purpose of analyzing both safety and efficacy. The promising results for potential virus inactivation within the breathing zone point to the overall potential of far‐UVC installations to prevent the spread of airborne diseases.

The film used in these studies was analyzed for suitability as a dosimeter through testing accuracy, angular response, wavelength response, and sources of uncertainty in film response. Overall, the results indicate these films are very good for this application. The angular response is in excellent agreement with a cosine response. A cosine response with electronic meters often requires diffusing optics, whereas this film can be used without additional complications. As with previous studies using film for UVC dosimetry,^20^ there is dramatic variation in film sensitivity across the UVC range. The wavelength dependence of the film requires that a unique calibration curve be obtained for a given source to be measured. This is evident by the two sources tested in this work, which are both filtered KrCl excimer lamps. The lamps having slightly different calibration curves is most likely due to the differences in filtering by the two manufacturers which is seen in the lamp spectrums (Figure S1). A weakness in using these films is that a minimum color change is necessary for the films to read accurately. The minimum exposure dose for the far‐UVC emitting lamps tested in this work was between 5 and 10 mJ/cm^2^ to achieve an accuracy of ±10%. Importantly this minimum exposure dose is dependent on the source being measured, so sources with more contributions from wavelengths in the 240 to 290 nm range will reach a minimum color change at a lower dose. It is therefore recommended that exposure times be adjusted for each film dosimetry situation to allow for sufficient color change to occur. Estimates of additional color change to the film from exposure to room lighting, time between exposure and scanning, and additional exposure to the film, if it is scanned multiple times, suggest that these factors are minimal in relation to the color change from typical UV exposures. Of the investigated sources of uncertainty, the most significant is the potential contribution to netCD from exposure to the room lighting. The dose–response accuracy results for the two lamps tested in this work (Figure 2) suggest that a minimum netCD of approximately 0.05 is necessary to estimate dose with a confidence of ±10%. We estimate that average room lighting could add an additional 0.0041 netCD in 2.3 h (the average sampling period used in this study) which equates to approximately a 12% dose increase for the lowest measured dose with a confidence of ±10% using the lamp calibrations from this study. That said, the intensity, duration, and spectrum of room lighting exposure will vary for each use case. If the films are used for prolonged dosimetry applications, then the inclusion of control exposures, where room lights are operating but the UV source is not, would help improve the accuracy of these films. Finally, it is important to note that the 2022 ACGIH TLVs specify that only exposure from a source subtending an angle of less than 80° at the detector is applicable for TLV measurement; therefore, future work to limit the film response to an 80° field of view would help conform to these recommendations.

There is significant opportunity for future work with films for far‐UVC dosimetry. The films are very inexpensive, only about $0.05 per cm^2^, compared to electronic meters which cost thousands of dollars each. Film dosimetry is also easy to implement with only minimal instruction for film handling and data recording. While evaluation requires image scanning and model fitting, the ease of shipment of exposed films for this analysis (procedures that were performed in this study) reduces this burden substantially. By using a high‐resolution flatbed scanner for color change quantification, only a very small film area is required to obtain dosimetry data. While a single pixel can theoretically be analyzed for dose, this study used films as small as 1 cm^2^ in this study to allow for ease of handling and to avoid measurements too close to an edge. Previous work examining variation in far‐UVC exposure across a manikin utilized multiple pieces of film distributed across the head during a single experiment,^16^ and a similar approach could be used for individual occupant monitoring with film cut to pieces of various sizes and shapes as desired. Such a study could also be useful to further elucidate the relationship between the magnitudes of skin and eye exposure doses within far‐UVC installations. Overall, the ability to make numerous measurements simultaneously with many pieces of film provides flexibility in evaluating exposures within real‐life installations of far‐UVC that are not feasible with electronic meters.

## Supporting information


Figures S1–S4
