# Supplementary material for: Film dosimetry for occupant exposure monitoring within Far‐UVC installations
Source: Photochem Photobiol. 2024 May 3;101(1):157–66. doi: 10.1111/php.13960 (PMC11737010; doi:10.1111/php.13960)
Supplement: Supplementary file 1 — Figures S1–S4 [file PHP-101-157-s001.pdf]

Film Dosimetry for Occupant Exposure Monitoring within Far-UVC Installations

David Welch[1], Raabia Hashmi[1], Camryn Petersen[1], Steven Erde[2], David J. Brenner[1], Ed Nardell[3]

[1] Center for Radiological Research, Columbia University Irving Medical Center, New York, NY

[2] College of Dental Medicine, Columbia University Irving Medical Center, New York, NY

[3] Division of Global Health Equity, Brigham & Women's Hospital, Harvard Medical School, Boston, MA

Supplemental Materials

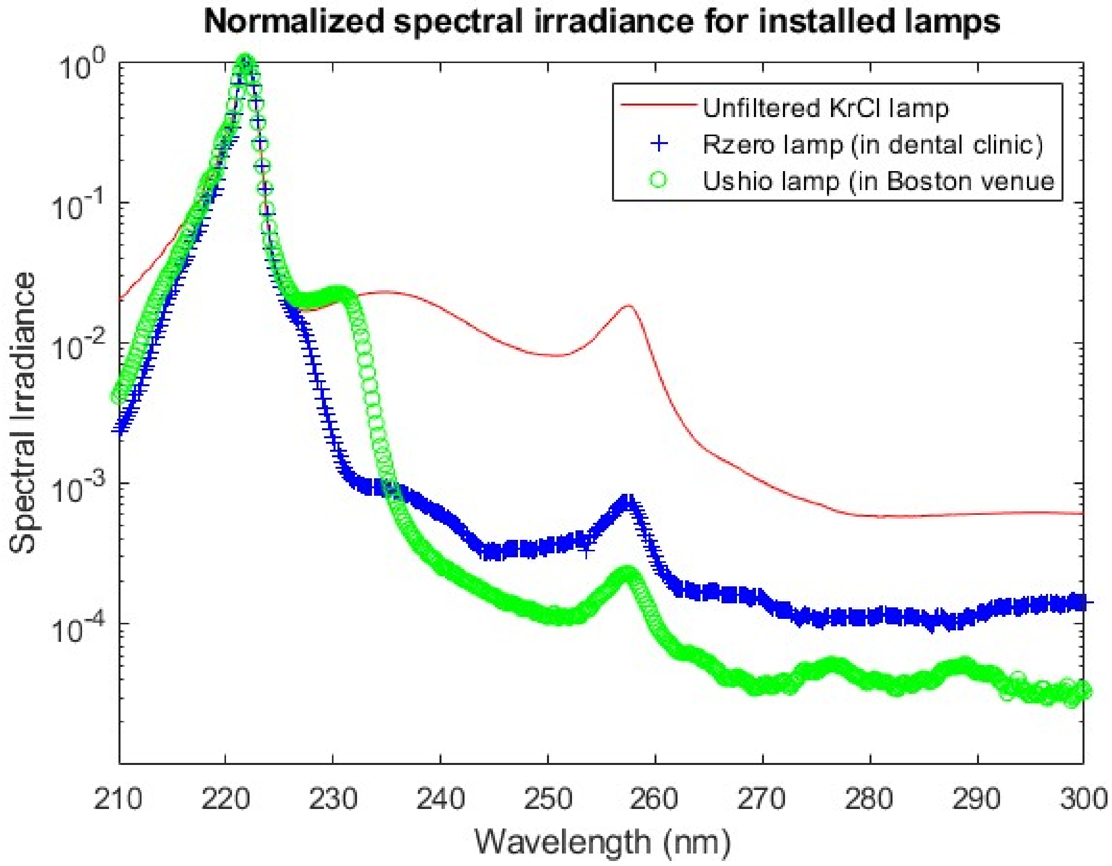

Figure S1. Normalized spectral irradiance for an unfiltered KrCl excimer lamp and the KrCl lamps with filters used in the two installations examined in this study.

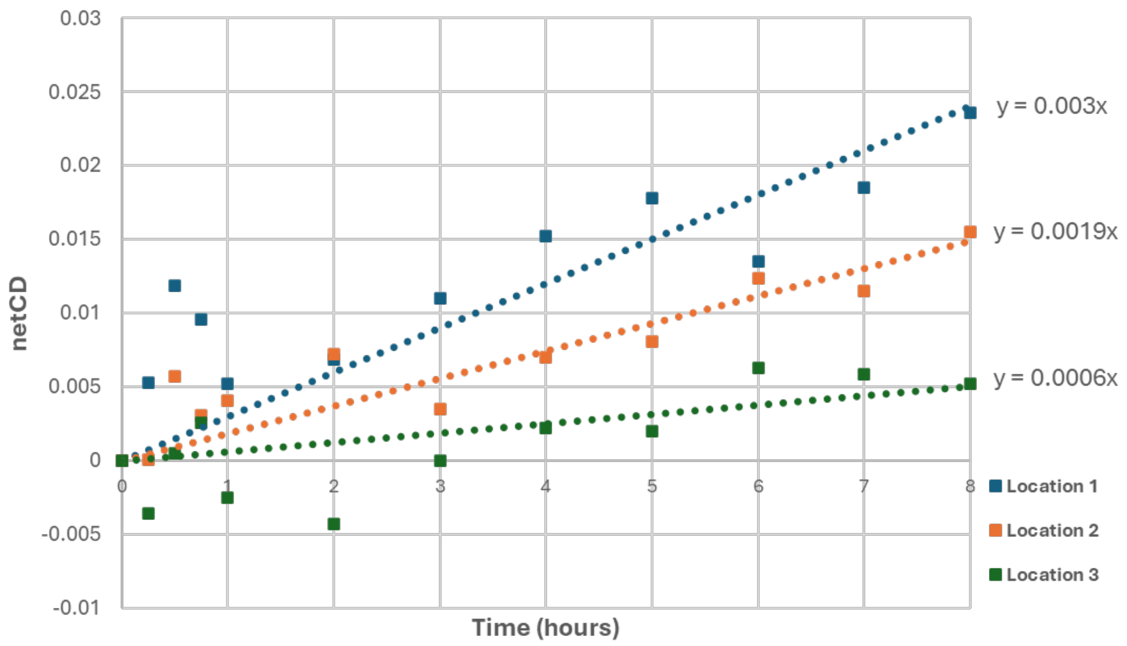

Figure S2. The effect of room lighting on the netCD of the film was examined at three locations with exposures of up to 8 hours. The average estimated increase to netCD was 0.0018 per hour.

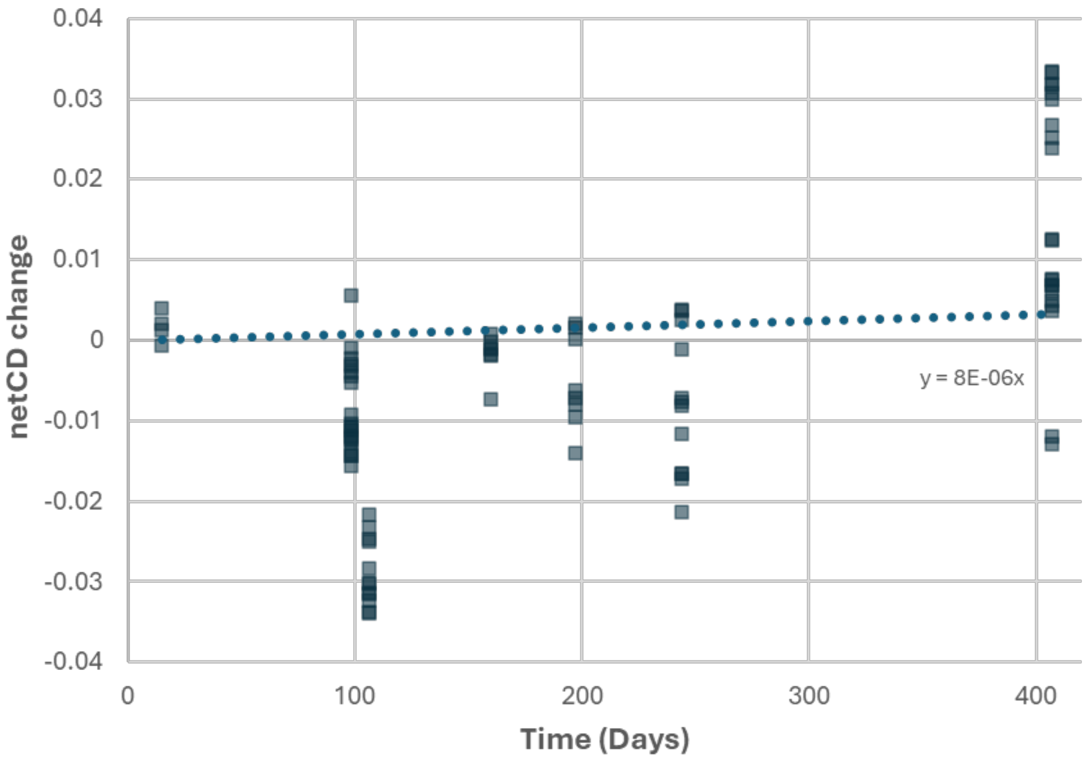

Figure S3. The netCD change for films shielded from exposure for various lengths of time between scans is plotted. The estimated change in netCD for time between scans is 8×10^{-6} per day.

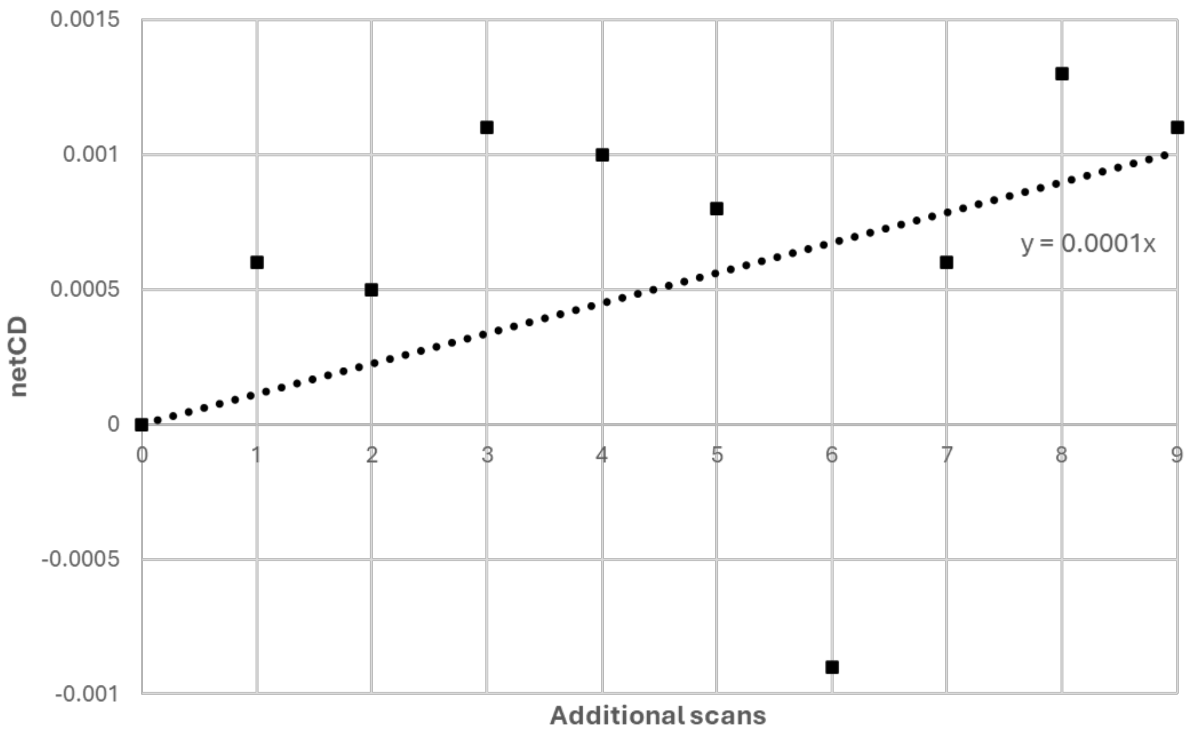

Figure S4. The effect of repeated scans of a film changing the netCD of a film is plotted for up to 9 additional scans. The estimated increase in netCD per scan is 1 x 10^{-4} per scan.
